# Carbohydrate Knowledge in People with Type 1 and Type 2 Diabetes in the NutriNet-Santé Cohort Study

**DOI:** 10.3390/nu18091415

**Published:** 2026-04-29

**Authors:** Sopio Tatulashvili, Alice Bellicha, Chantal Julia, Laurent Bourhis, Nathalie Arnault, Hélène Bihan, Serge Hercberg, Mathilde Touvier, Michael Joubert, Emmanuel Cosson

**Affiliations:** 1 AP-HP, Department of Endocrinology-Diabetology-Nutrition, Avicenne Hospital, Université Sorbonne Paris Nord, CINFO, CRNH-IDF, 93017 Bobigny, France; 2 Equipe de Recherche en Epidémiologie Nutritionnelle (EREN), Université Sorbonne Paris Nord and Université Paris Cité, INSERM, INRAE, CNAM, Center of Research in Epidemiology and StatisticS (CRESS), 93017 Bobigny, France; a.bellicha@eren.smbh.univ-paris13.fr (A.B.);; 3 Health Education Laboratory EA-3412, Universite Sorbonne Paris Nord, 93017 Bobigny, France; 4 Diabetes Care Unit, Caen University Hospital, UNICAEN, 14000 Caen, France

**Keywords:** type 1 diabetes, type 2 diabetes, carbohydrate counting knowledge, Nutri-Net Santé cohort, GluciQuizz questionnaire

## Abstract

**Background:** Effective glycemic control in diabetes management relies heavily on dietary carbohydrate knowledge. This study aimed to assess carbohydrate knowledge in individuals with type 1 diabetes (T1D) and insulin-treated type 2 diabetes (itT2D) using the GluciQuizz tool. **Methods:** A total of 465 persons (96 with T1D, 153 with itT2D; 89 and 127 matched controls without diabetes, respectively) from the French NutriNet-Santé prospective cohort were included. Participants completed the GluciQuizz questionnaire, which evaluates carbohydrate knowledge across five domains: carbohydrate food recognition; carbohydrate food content; nutrition label reading; glycemic targets and hypoglycemia prevention and treatment; and carbohydrate content of meals. **Results:** The mean age ± standard deviation of participants with diabetes was 65.8 ± 11.2 years, 44.2% male, with a diabetes duration of 23.3 ± 12.9 years. T1D participants scored significantly higher on the GluciQuizz compared to those with itT2D (23.9 ± 5.0 vs. 17.5 ± 5.6, *p* < 0.001). In secondary analysis, T1D participants showed superior knowledge to their matched controls without diabetes, whereas itT2D participants showed similar knowledge to their matched controls without diabetes. **Conclusions:** T1D participants demonstrated the best carbohydrate knowledge compared to those with itT2D. Targeted educational interventions in itT2D populations may improve dietary management and clinical outcomes.

## 1. Introduction

Diabetes is a metabolic disorder characterized by hyperglycemia, resulting from disturbances in insulin secretion, insulin action, or both [1]. Type 1 diabetes (T1D) is an autoimmune disease marked by the destruction of insulin-producing beta cells, accounting for 5 to 10% of all diabetes mellitus cases, and leading to a significant deficiency in insulin secretion by the pancreas [2,3]. It is managed with exogenous insulin, which fulfills the insulin required for survival (approximately 50% of the need) and the insulin needed for food intake (the remaining 50%) [3]. Type 2 diabetes (T2D) represents the main diabetes type. It is characterized by insulin resistance and relative insulin deficiency [4]. Initially, endogenous insulin secretion can be stimulated; however, after a few years, exogenous injectable insulin therapy could become necessary [2,3].

Glycemic control in patients with diabetes is critical as it directly impacts the development of long-term diabetic complications [5,6,7,8]. Achieving glycemic control in diabetes treated with insulin has become more feasible with recent technological advances, such as continuous glucose monitoring (CGM) systems [9], continuous subcutaneous insulin infusion (CSII) [10,11], and automated insulin delivery (AID) systems for T1D [12,13]. Despite these advancements, postprandial glucose—generally defined as the glucose level two hours after the beginning of a meal—remains a significant challenge. Indeed, postprandial hyperglycemia is still very common [9,14] and is considered an independent contributor to diabetes complications [15].

The carbohydrate content of a meal is one of the most important factors influencing postprandial glucose levels and can be controlled by individuals. Indeed, carbohydrate nutritional knowledge plays a key role in optimizing postprandial glucose control because it helps individuals with diabetes to adapt their diet and, when appropriate, to titrate their prandial insulin [16]. The meta-analysis by Vaz et al. provides evidence favoring the use of carbohydrate counting in the management of adult patients with T1D [17]. Carbohydrate counting is also an effective, low-cost tool for reducing HbA1c and glycemic variability in individuals with multiple daily insulin-treated T2D, without an associated increase in hypoglycemia or body mass index (BMI) [18].

Beyond its role in postprandial glucose control, carbohydrate counting serves as a meal-planning tool that allows significant variation in food choices for individuals with diabetes [19], thereby providing flexibility in food intake [20]. With fewer dietary restrictions and the ability to decide the number of meals, carbohydrate counting may improve disease acceptance and overall quality of life [21].

Nutritional education on carbohydrate counting is offered to patients, enabling them to calculate their mealtime insulin dose based on the quantity of carbohydrates consumed and their individual insulin sensitivity [22]. However, to individualize and optimize the educational process, the carbohydrate-counting knowledge of patients needs first to be evaluated. Currently, few questionnaires exist worldwide for assessing the carbohydrate-counting knowledge of individuals living with diabetes, whether they are children and parents [23,24] or adults [25,26]. The AusPCQ [24] is an adaptation of the PedCarbQuiz [23] specifically designed for Australian children and parents. Similarly, the Singapore questionnaire [26] also uses the structure of the PedCarbQuiz [23] but has modified the meal content to suit the Asian adult population. The AdultCarbQuiz, produced and validated in the United States of America (USA) by Watts et al. [25], was designed for Western adults but was not well-adapted to French–European nutritional habits. In this context, our teams translated it into French, adapted it to French nutritional habits, and validated it as the GluciQuizz self-administered questionnaire [27] (Supplementary Materials). 

The aim of this study is to evaluate carbohydrate knowledge using the GluciQuizz questionnaire among French people living with T1D and insulin-treated T2D and their respective controls without diabetes within the French cohort NutriNet-Santé. We also aimed to explore associations with dietary intake.

## 2. Methods and Data

### 2.1. NutriNet-Santé Cohort

The present study used observational data from the NutriNet-Santé study. The NutriNet-Santé study is an Internet-based cohort launched in May 2009 [28]. Its purpose is to study the determinants of diets, nutritional status, and physical activity as well as their associations with health. The participants, recruited on a voluntary basis, are French-speaking adults with access to the Internet. Participants are invited to provide detailed information using a personal account on the study website (https://etude-nutrinet-sante.fr/, accessed on 5 March 2026). Five questionnaires are proposed at inclusion to collect information about sociodemographic and lifestyle data (e.g., birthdate, sex, education level, smoking status) [29], anthropometric data (e.g., self-reported height, body weight) [30,31], health status (e.g., personal and family medical history, medical treatments), and dietary habits (three non-consecutive 24 h dietary records). After inclusion, participants are required to update their data by completing annual (all five questionnaires) or biannual (health, weight, and dietary) questionnaires. Additionally, targeted questionnaires on specific research topics are administered regularly.

T1D and T2D were detected with a multi-source approach. Throughout follow-up, participants could report health events, medical treatment, and examinations via the biannual health questionnaires or at any time directly via the health interface of their personal account. Participants are asked to declare all currently taken medications and treatments via the check-up and yearly questionnaires. A search engine with an embedded exhaustive Vidal^®^ drug database is used to facilitate medication data entry for the participants. Medical information was reviewed by NutriNet-Santé physician experts to ascertain diabetes cases. Moreover, the NutriNet-Santé cohort was linked to the national health insurance system database (Systeme National d’Information Inter-regimes de l’Assurance Maladie [SNIIRAM]) to collect additional information about medical treatments and consultations.

The NutriNet-Santé study is in line with the principles of the Helsinki Declaration [32], and the protocol has been approved by both the INSERM Ethical Evaluation Committee (CEEI) (no. 0000388FWA00005831) and the National Committee for Information Technology and Freedom (CNIL) (nos. 908450 and 909216). Electronic informed consent was obtained from all participants. The study is registered in ClinicalTrials.gov (NCT03335644).

### 2.2. Selection of Participants

In February 2023, all T1D and insulin-treated T2D cases who completed at least two NutriNet-Santé questionnaires during the last 3 years were invited to participate in this study. Lately, in August 2023, matched controls with no diabetes were invited to participate in this study. Matching criteria were sex (male or female), age (exact matching was performed for the age on 20 February 2023), BMI (accepting a difference of ±2 points in BMI), education level (exact matching was performed for the following education levels: less than high school, high school diploma or equivalent, graduate degree and others), and region of residence in France (exact matching was performed for the following regions: North, East, West, South and Other).

Eligible participants were sent an email asking them to complete the GluciQuizz questionnaire assessing carbohydrate knowledge. Alongside the GluciQuizz questionnaire, participants were asked additional questions about their current weight and, if relevant, about type of diabetes, year of diabetes diagnosis, diabetes treatment, and most recent hemoglobin A1c (HbA1c) levels.

We then selected the participants who were certainly qualified with T1D and insulin-treated T2D and who completed the GluciQuizz questionnaire.

### 2.3. Assessment of Carbohydrate Knowledge

GluciQuizz is a self-administered questionnaire that has been adapted from the American self-administered questionnaire AdultCarbQuiz [25] and validated for French patients [27]. This questionnaire (Annex 1) includes 36 items and evaluates the following domains of carbohydrate knowledge: domain 1, carbohydrate food recognition (13 items); domain 2, carbohydrate food content (7 items); domain 3, nutrition label reading (4 items); domain 4, glycemic targets and hypoglycemia prevention and treatment (8 items); and domain 5, carbohydrate content of meals (4 items). The maximum score for the GluciQuizz questionnaire, i.e., 36 points, corresponds to 1 point for each correct response. Participants with diabetes were invited to complete all domains of the questionnaire, whereas control participants were asked to complete only domains 1, 2 and 3; given that domains 4 and 5 are specific to participants with diabetes. The questionnaire is available here: https://info.etude-nutrinet-sante.fr/upload/siteinfo/protectednew/Quest_GluciQuizz.pdf (accessed on 5 March 2026).

### 2.4. Anthropometric and Dietary Data Collection

BMI was calculated based on the self-reported weight and height with the following formula: BMI = weight (kg)/height (m)^2^. We used the most recent weight available reported during the last three years of follow-up. Participants also provided their current weight at the time of completing the GluciQuizz questionnaire, which was cross-checked with the cohort data [30,31].

At inclusion and every 6 months thereafter, NutriNet-Santé participants are invited to fill out three validated non-consecutive days of 24 h dietary records, randomly assigned over a 2-week period, including two weekdays and one weekend day (to account for variability in the diet across the week and the seasons). Energy and nutrient intakes are computed using the NutriNet-Santé composition table containing 3500 generic food/beverage items and mixed dishes for which standard French recipes have been defined by nutrition professionals [33,34,35,36]. For our study, we used the data from two dietary assessment periods (a total of six days of dietary records) reported for three years preceding the GluciQuizz questionnaire. We then evaluated the total energy intake (Kcal/day) and the percentage of energy from carbohydrates, protein and fat. We evaluated fiber intake. We then detailed several variables concerning carbohydrates such as average dietary glycemic index, dietary glycemic load (g/d), carbohydrates from low glycemic index foods (glycemic index 0 to 39), carbohydrates from medium/high glycemic index foods (medium glycemic index 40 to 59, high glycemic index 60 to 100), added sugar (g/d), fruits (g/d), sweet/sweetened foods except for fruit (g/d), 100% fruit juice (mL/d), and sweet/sweetened beverages except for 100% fruit juice (mL/d).

### 2.5. Outcomes

The primary aim was to compare carbohydrate knowledge between individuals with T1D and those with insulin-treated T2D, based on the overall GluciQuizz score (primary outcome) and the scores for each domain individually (secondary outcomes).

The secondary aims included the following:

1. Comparing carbohydrate knowledge, based on the domains 1 (carbohydrate food recognition), 2 (carbohydrate food content) and 3 (nutrition label reading) of the GluciQuizz score, between individuals with T1D and their matched controls without diabetes, or between insulin-treated T2D and their matched controls.
2. Assessing dietary carbohydrate intake among individuals with T1D or insulin-treated T2D, stratified by their respective median overall GluciQuizz score, to explore potential associations between carbohydrate knowledge and dietary behaviors.

### 2.6. Statistical Analysis

The data collected were described using frequency and percentage for categorical variables. Mean and standard deviation (SD) were used for quantitative variables. 

To compare characteristics between T1D and T2D patients, as well as between participants with GluciQuizz scores above and below the median within each diabetes group, appropriate univariate tests were applied. Fisher’s exact test or the chi-squared test was used for categorical variables, while the Mann–Whitney U test was employed for quantitative variables due to the non-normal distribution. We also performed a subgroup analysis to compare characteristics between T1D and T2D participants specifically on multiple daily (basal-bolus) insulin therapy.

Additionally, to compare diabetes participants and their matched controls, paired statistical tests were used, including McNemar’s test for categorical variables and the Wilcoxon signed-rank test for quantitative variables.

To evaluate differences in total GluciQuizz scores (five domains, maximum: 36 points) between T1D and T2D participants, a univariate linear regression model was initially applied. We then performed a multivariable linear regression model adjusting for relevant covariates: age, sex, BMI, education level and region of France. A univariate linear regression model was also performed separately for each of the five domains of the GluciQuizz.

Additionally, we used a mixed-effects linear model to compare the sum of scores from the first three GluciQuizz domains (maximum: 24 points) between T1D and T2D patients and their specific matched controls. Participant pairs were treated as random effects to account for within-pair dependencies.

To satisfy the assumptions of linear regression models, GluciQuizz scores were normalized using ordered quantile normalization transformation, ensuring a Gaussian distribution of residuals. All statistical analyses were performed using R software (Version 3.6.1). A *p*-value of <0.05 was considered statistically significant.

## 3. Results

### 3.1. Participant Characteristics

There were 465 participants. We included 249 participants with diabetes (96 with T1D, 153 with insulin-treated T2D). The characteristics of all participants with T1D and insulin-treated T2D are presented in Table 1. Participants with T1D, as compared to T2D, were more often female, 9 years younger, and their BMI was 6 kg/m^2^ lower. Additionally, the HbA1c of participants with T1D was 0.2% lower than that of the participants with T2D. Their insulin regimen differed: only 27% of participants with T2D were treated with multiple daily insulin injections, while the remaining 73% received basal insulin. Participants with T2D reported a significantly higher prevalence of cardiovascular complications and cardiovascular risk factors (including hypertension, dyslipidemia, obesity, smoking history, and family history of cardiovascular disease) than participants with T1D. Moreover, the educational level of the two groups significantly differed, with higher education in the participants with T1D.

Table 2 compares the 89 participants with T1D who had specific controls without diabetes and 127 participants with T2D and their specific controls. Participants with T1D were more prone to have hypercholesterolemia and hypertension than their controls. Participants with T2D compared to their controls reported more familial history of diabetes and cardiovascular disease, more personal cardiovascular disease and higher BMI, hypercholesterolemia, hypertriglyceridemia and hypertension.

### 3.2. GluciQuizz Scores by Diabetes Type

**Figure 1:**
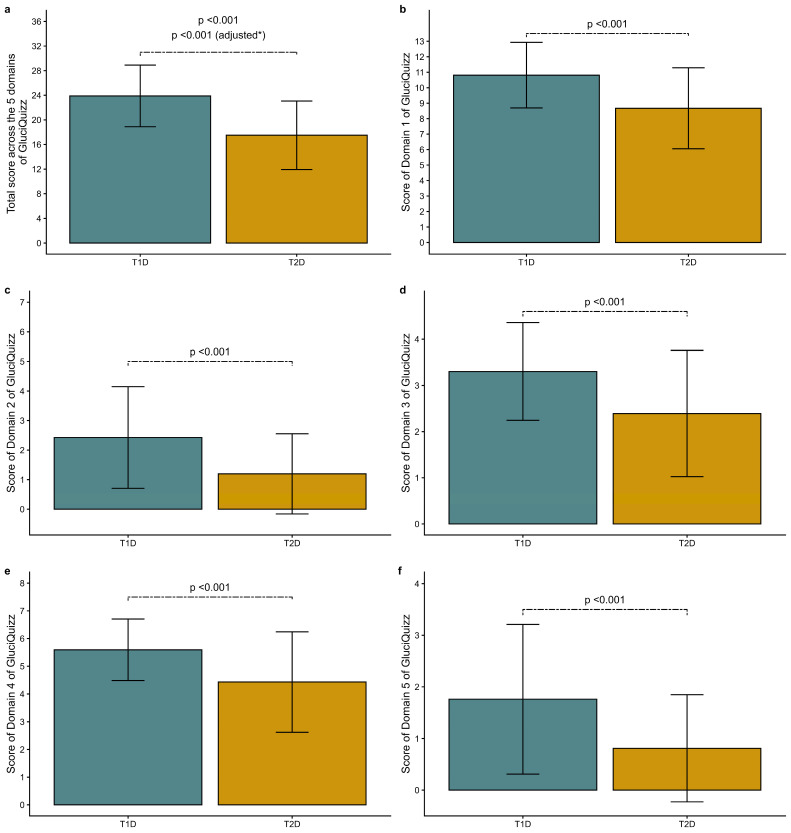
GluciQuizz scores for participants with type 1 (T1D) and type 2 diabetes (T2D). * Adjusted for age, sex, body mass index, education level and region of France. The *p*-value is obtained from the linear model. Values are presented as mean ± SD. (**a**) shows the total score of the 5 domains of GluciQuizz (maximum 36); (**b**) shows the score of domain 1 of GluciQuizz (carbohydrate food recognition, max 13 points); (**c**) shows the score of domain 2 of GluciQuizz (carbohydrate food content, max 7 points); (**d**) shows the score of domain 3 of GluciQuizz (nutrition label reading, max 4 points); (**e**) shows the score of domain 4 of GluciQuizz (glycemic targets and hypoglycemia prevention and treatment, max 8 points); (**f**) shows the score of domain 5 of GluciQuizz (carbohydrate content of meals, max 4 points).

In the univariate linear model, T1D participants had a significantly higher mean (±SD) total score (23.9 ± 5.0) compared to T2D participants (17.5 ± 5.6, *p* < 0.001) (Figure 1a), and this difference was consistently observed across all five domains (Figure 1b–f). In the multivariable model, total score was still significantly higher for T1D participants. A subgroup analysis of participants with either T1D or T2D who were on a basal-bolus insulin regimen (27% of T2D) showed similar trends in both the overall and individual domain scores (Supplementary Table S1).

### 3.3. GluciQuizz Scores in the Patients with Diabetes vs. Their Controls

**Figure 2:**
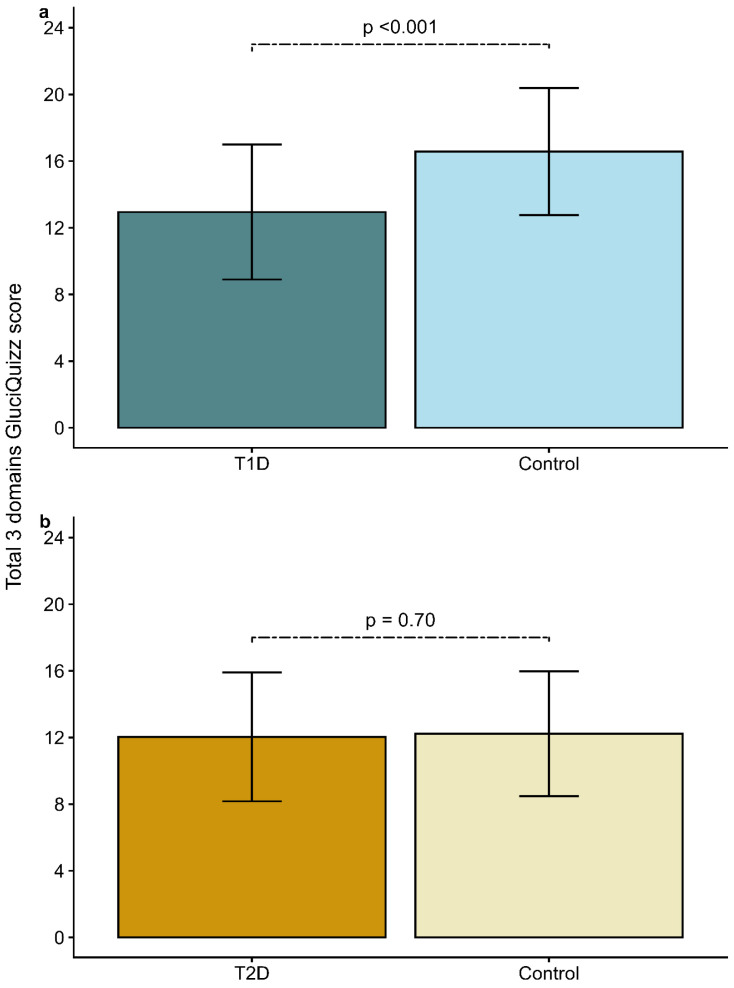
Total of the scores of the 3 first domains of GluciQuizz for (**a**) participants with type 1 diabetes (T1D) and their matched controls and (**b**) participants with type 2 diabetes (T2D) and their matched controls. Values are presented as mean ± SD. Controls for T1D and then T2D were matched for age, sex, body mass index, education level and region of France. The maximum total score for the 3 first domains was 24 points, considering domain 1 (carbohydrate food recognition, max 13 points), domain 2 (carbohydrate food content, max 7 points) and domain 3 (nutrition label reading, max 4 points).

Compared with their matched controls without diabetes (Table 2), T1D participants showed significantly higher GluciQuizz scores overall and for domains 1 and 2, but not for domain 3 (Figure 2a). GluciQuizz scores were similar in patients with T2D and their controls (Figure 2b).

### 3.4. Total Energy and Carbohydrate Intake and GluciQuizz Results in Participants with Diabetes

Dietary data were available for 66 T1D participants who were separated into two groups based on their median Gluciquizz score (i.e., 25). As shown in Table 3, only the average proportion of daily energy from fat was higher in those with the highest GluciQuizz score. 

Similarly, dietary data were available for 106 T2D participants, who were also separated according to their median Gluciquizz score (i.e., 18). Table 3 shows that participants with higher GluciQuizz scores consumed more fiber, added sugars and sweet/sweetened foods compared to those with lower scores. 

## 4. Discussion

The findings of our study highlight significant disparities in carbohydrate knowledge between T1D and insulin-treated T2D populations. Individuals with T1D had higher GluciQuizz scores compared to individuals with insulin-treated T2D as well as their matched controls, while GluciQuizz scores did not differ between individuals with T2D and their matched controls. Surprisingly, insulin-treated T2D individuals with higher GluciQuizz scores consumed more added sugar and sweet/sweetened food. 

### 4.1. Disparities in Carbohydrate Knowledge

The significantly higher GluciQuizz scores observed in T1D participants compared to T2D participants underscore fundamental differences in the emphasis placed on carbohydrate counting in their respective management strategies. Carbohydrate counting is an integral part of intensive insulin therapy in T1D, where precise carbohydrate estimation is required to calculate insulin doses. This practice fosters a deeper understanding of carbohydrate recognition, content estimation, and overall dietary control [22]. In contrast, T2D management often focuses on broader dietary guidelines, including portion control, glycemic index, and reduction in total carbohydrate intake, rather than specific carbohydrate counting [37]. While an educational program on carbohydrate counting begins early in patients with T1D, it usually begins when insulin therapy, and especially prandial insulin, is initiated in patients with T2D. That is the reason why we only included insulin-treated patients with T2D in the present study. However, the exact duration of insulin treatment for individuals with T2D was unavailable in our study. However, since insulin therapy is typically introduced several years after diagnosis in T2D, we suggest that these individuals have a shorter duration of insulin treatment and, consequently, less time for therapeutic education. However, the sensitivity analysis considering only individuals with T1D and T2D treated with multiple daily injections showed similar results. Additionally, it is worth noting that in our study, individuals with T1D had a higher education level compared to those with T2D, which may potentially explain their adherence to educational programs. However, we adjust our results for education level. 

Interestingly, T2D participants did not score significantly higher than their matched controls without diabetes. This finding suggests that standard educational interventions for insulin-treated T2D patients may not adequately address carbohydrate knowledge, highlighting a gap in current diabetes education practices in France. Structured educational programs tailored to T2D patients have been shown to improve carbohydrate knowledge, glycemic control, and dietary flexibility [18]. Therefore, implementing more focused carbohydrate education in T2D care may yield substantial benefits. On the other hand, these results could partly be explained by the greater dietary interest of the NutriNet cohort participants, indicating a good level of carbohydrate knowledge among those without diabetes. 

### 4.2. Role of Educational Interventions

Education is a critical component of diabetes self-management. For T1D, structured programs such as Dose Adjustment for Normal Eating (DAFNE) have demonstrated success in improving carbohydrate knowledge and enabling effective insulin dose adjustments, ultimately reducing HbA1c without increasing hypoglycemia risk [17]. The results of this study reflect the benefits of such structured approaches in T1D populations, as evidenced by their higher GluciQuizz scores.

However, educational interventions in insulin-treated T2D often lack the same focus on carbohydrate-specific knowledge, instead emphasizing weight loss, calorie reduction, and portion control [37]. This generalized approach may explain the lower scores observed in T2D participants, particularly in domains requiring specific carbohydrate identification or estimation. Enhancing insulin-treated T2D education with carbohydrate-specific components could not only improve knowledge but also promote a better quality of life by allowing greater freedom in dietary behavior, ultimately leading to better glycemic outcomes.

The role of carbohydrate knowledge in improving glycemic outcomes has been well-documented, particularly in T1D [17]. Accurate carbohydrate counting enables precise insulin dose adjustments, reducing HbA1c and glycemic variability [17]. The slightly lower HbA1c levels observed in T1D participants in this study may reflect the benefits of superior carbohydrate knowledge, as indicated by their higher GluciQuizz scores.

For T2D, the relationship between carbohydrate knowledge and glycemic control is less direct, given the heterogeneity in disease progression, treatment regimens, and dietary strategies. While carbohydrate education has shown efficacy in improving outcomes for T2D patients on intensive insulin therapy [18], its impact on those on oral treatment or on basal insulin therapy does not differ from standard dietary care [38]. Further research is needed to determine how enhanced carbohydrate education can be tailored to meet the diverse needs of T2D populations.

### 4.3. Associations with Dietary Behavior

In T1D participants, the average proportion of daily energy from fat was higher in those with GluciQuizz scores above the median (≥25) without a difference in overall energy intake. This result might correspond to a relative reduction in carbohydrate consumption, even if the observed difference in carbohydrate intake was not statistically significant, while protein intake remained stable. This raises questions about the balance of macronutrient intake in T1D patients with advanced carbohydrate knowledge and highlights the need for comprehensive nutritional education addressing overall dietary patterns, not just carbohydrate counting.

In T2D participants, those with higher GluciQuizz scores (≥18) demonstrated increased fiber intake alongside higher consumption of added sugars and sweet/sweetened foods. This paradox could suggest that better carbohydrate knowledge does not always translate into healthier overall dietary habits in T2D patients. Conversely, the increased intake of sweetened foods and added sugars may reflect a sense of greater dietary freedom enabled by better carbohydrate knowledge, as these individuals may feel more confident in managing occasional indulgences within the context of overall glycemic control.

### 4.4. Strengths and Limitations

This study benefits from the use of a validated tool (GluciQuizz) to assess carbohydrate knowledge comprehensively across multiple domains. The inclusion of matched controls strengthens the comparative analysis by reducing potential confounding factors, thereby enhancing the reliability of the findings. Furthermore, the study’s large and well-characterized cohort adds statistical power and improves generalizability to the results, offering robust insights.

However, several limitations should be considered. First, the reliance on self-reported dietary data may introduce recall bias; these results should be interpreted as exploratory. Second, the cross-sectional design precludes causal inference, limiting the ability to establish direct relationships between carbohydrate knowledge and glycemic outcomes. Third, while the GluciQuizz captures multiple aspects of carbohydrate knowledge, it does not assess other key components of diabetes self-management, such as carbohydrate counting and medication adherence, which may also influence glycemic control. As in other studies investigating health and diet in which people enroll voluntarily, this study included participants with a higher educational level and healthier lifestyles than the general French population [39] and maybe high nutritional literacy. Finally, this questionnaire was developed for T1D and, more specifically, for patients with T1D practicing flexible insulin therapy. Therefore, its validity for T2D and for individuals without diabetes is uncertain. Therefore, caution is needed in generalizing the findings.

### 4.5. Future Directions

Future research should explore the longitudinal impact of carbohydrate education on glycemic control, quality of life, and complication rates. Investigating the role of digital tools and mobile applications in delivering carbohydrate education offers another promising avenue for improving accessibility and engagement [40,41]. Our team is exploring the relationship between carbohydrate knowledge, ambulatory glucose profiles, and diabetes complications across different modes of insulin therapy in individuals living with T1D. The study will be published soon. We are also interested in comparing carbohydrate knowledge and use of the Nutri-Score [42,43] among the people with T1D, insulin-treated T2D and controls without diabetes, as well as its impact on shopping baskets.

## 5. Conclusions

The disparities in carbohydrate knowledge between T1D and insulin-treated T2D participants underscore the need for tailored educational strategies to bridge these gaps. While T1D participants benefit from structured carbohydrate education as part of their management, insulin-treated T2D populations may similarly benefit from such approaches to improve dietary knowledge and probably glycemic outcomes. However, these individual patient empowerment measures should not replace the need for positive changes in the food environment. This includes regulating marketing and advertising of sugary products, implementing standardized Nutri-Score labeling, securing industry agreements for product reformulation and sugar reduction, and adopting pricing policies to make healthier, lower-sugar products more accessible.

## Figures and Tables

**Table 1:** General characteristics of participants with type 1 (T1D) and type 2 diabetes (T2D).

General Characteristics	Total *N* = 249 Patients	T1D *N* = 96 Patients	T2D *N* = 153 Patients	*p*	Avalable Data (*N*)
**Age** years	65.8 ± 11.2	60.3 ± 12.6	69.3 ± 8.8	<0.001	249
**Sex**, male	110 (44.2%)	30 (31.2%)	80 (52.3%)	0.002	249
**Region**				>0.9	249
North	92 (36.9%)	38 (39.6%)	54 (35.3%)		
South	56 (22.5%)	20 (20.8%)	36 (23.5%)		
East	51 (20.5%)	19 (19.8%)	32 (20.9%)		
West	47 (18.9%)	18 (18.8%)	29 (19.0%)		
Other (other and overseas)	3 (1.2%)	1 (1.0%)	2 (1.3%)		
**Body mass index**, kg/m^2^	28.2 ± 6.2	24.5 ± 5.5	30.6 ± 5.4	<0.001	249
**Diabetes duration**, years	23.3 ± 12.9	25.2 ± 15.7	22.0 ± 10.7	0.08	248
**HbA1c**	7.1 ± 0.9	7.0 ± 0.7	7.2 ± 1.0	0.03	224
**Treatment of diabetes**				<0.001	249
Single injection	106 (42.6%)	7 (7.3%)	99 (64.7%)		
Multiple daily injection	94 (37.8%)	52 (54.2%)	42 (27.5%)		
Continuous subcutaneous insulin infusion with or without closed loop	49 (19.7%)	37 (38.5%)	12 (7.8%)		
**Complications**					
**Cardiovascular or neurovascular events**	51 (20.5%)	8 (8.3%)	43 (28.1%)	<0.001	249
**Hypertension**	156 (62.7%)	38 (39.6%)	118 (77.1%)	<0.001	249
**Hypercholesterolemia**	144 (57.8%)	39 (40.6%)	105 (68.6%)	<0.001	249
**Hypertriglyceridemia**	66 (26.5%)	10 (10.4%)	56 (36.6%)	<0.001	249
**Smoking status**				0.003	240
Never smoker	84 (35.0%)	43 (47.8%)	41 (27.3%)		
Former smoker	142 (59.2%)	41 (45.6%)	101 (67.3%)		
Current smoker	14 (5.8%)	6 (6.7%)	8 (5.3%)		
**Alcohol use**, g ethanol/d	5.2 ± 8.3	5.4 ± 6.8	5.0 ± 9.1	0.70	231
**Family history**					
Diabetes	140 (56.2%)	39 (40.6%)	101 (66.0%)	<0.001	249
Cardio-vascular diseases	215 (86.3%)	75 (78.1%)	140 (91.5%)	0.005	249
Obesity	76 (30.5%)	18 (18.8%)	58 (37.9%)	0.002	249
**Education level**				<0.001	249
Less than high school	66 (26.5%)	14 (14.6%)	52 (34.0%)		
High school diploma or equivalent	105 (42.2%)	39 (40.6%)	66 (43.1%)		
Graduate degree	69 (27.7%)	41 (42.7%)	28 (18.3%)		
Other	9 (3.6%)	2 (2.1%)	7 (4.6%)		
**Retired**	172 (69.1%)	48 (50.0%)	124 (81.0%)		

Data are *N* (%) or mean ± standard deviation.

**Table 2:** Characteristics of participants with type 1 (T1D) and type 2 diabetes (T2D) and their matched controls without diabetes.

General Characteristics	T1D	Matched * Controls Without T1D	*p*	T2D	Matched * Controls Without T2D	*p*
	*N* = 89	*N* = 89		*N* = 127	*N* = 127	
**Age** years	60.7 ± 12.2	60.8 ± 12.2	0.29	69.0 ± 8.8	69.1 ± 8.6	0.56
**Sex**, male	28 (31.5%)	28 (31.5%)	>0.9	71 (55.9%)	71 (55.9%)	>0.9
**Region**			>0.9			>0.9
North	33 (37.1%)	33 (37.1%)		49 (38.6%)	49 (38.6%)	
South	20 (22.5%)	20 (22.5%)		26 (20.5%)	26 (20.5%)	
East	19 (21.3%)	19 (21.3%)		25 (19.7%)	25 (19.7%)	
West	16 (18.0%)	16 (18.0%)		26 (20.5%)	26 (20.5%)	
Other (other and overseas)	1 (1.1%)	1 (1.1%)		1 (0.8%)	1 (0.8%)	
**Body mass index**, kg/m^2^	24.2 ± 4.7	24.1 ± 4.6	0.77	29.3 ± 4.7	29.1 ± 4.6	0.009
**Cardiovascular or neurovascular events**	7 (7.9%)	6 (6.7%)	>0.9	32 (25.2%)	15 (11.8%)	0.01
**Hypercholesterolemia**	37 (41.6%)	15 (16.9%)	<0.001	85 (66.9%)	40 (31.5%)	<0.001
**Hypertriglyceridemia**	8 (9.0%)	3 (3.4%)	0.21	45 (35.4%)	13 (10.2%)	<0.001
**hypertension**	36 (40.4%)	16 (18.0%)	0.002	95 (74.8%)	54 (42.5%)	<0.001
**Smoking status**			0.24			0.13
Never smoker	40 (47.6%)	39 (44.3%)		31 (24.8%)	42 (33.3%)	
Former smoker	38 (45.2%)	47 (53.4%)		86 (68.8%)	81 (64.3%)	
Current smoker	6 (7.1%)	2 (2.3%)		8 (6.4%)	3 (2.4%)	
**Alcohol use**, g ethanol/d	5.7 ± 7.0	4.9 ± 6.2	0.42	5.0 ± 8.8	7.3 ± 9.2	0.05
**Family history**						
Diabetes	35 (39.3%)	23 (25.8%)	0.08	83 (65.4%)	34 (26.8%)	<0.001
Cardio-vascular diseases	70 (78.7%)	69 (77.5%)	>0.9	115 (90.6%)	103 (81.1%)	0.04
Obesity	17 (19.1%)	15 (16.9%)	0.84	43 (33.9%)	43 (33.9%)	>0.9
**Education level**			>0.9			0.54
Less than high school <bac	12 (13.5%)	13 (14.6%)		46 (36.2%)	44 (34.6%)	
High school diploma or equivalent	36 (40.4%)	36 (40.4%)		51 (40.2%)	58 (45.7%)	
Graduate degree	39 (43.8%)	39 (43.8%)		23 (18.1%)	22 (17.3%)	
Other	2 (2.2%)	1 (1.1%)		7 (5.5%)	3 (2.4%)	
Retired	46 (51.7%)	46 (51.7%)		101 (79.5%)	101 (79.5%)	
**GluciQuizz ^**						
Total 3 domains (max 24)	16.6 ± 3.8	12.9 ± 4.0	<0.001	12.2 ± 3.7	12.0 ± 3.9	0.70
Domain 1 (max 13)	10.8 ± 2.2	8.6 ± 2.7	<0.001	8.7 ± 2.5	8.3 ± 2.8	0.23
Domain 2 (max 7)	2.4 ± 1.7	1.1 ± 1.4	<0.001	1.1 ± 1.4	1.0 ± 1.1	0.70
Domain 3 (max 4)	3.3 ± 1.0	3.2 ± 1.1	0.50	2.4 ± 1.4	2.7 ± 1.2	0.06

Data are *N* (%) or mean ± standard deviation. * Matched for age, sex, body mass index, education level and region of France. ^ *p*-value for the total GluciQuizz score and for each domain is obtained from the mixed-effects linear model.

**Table 3:** Comparing the carbohydrate intake according to GluciQuizz score: ≤median vs. >median, for participants with type 1 (T1D) and type 2 diabetes (T2D).

Stratified on Median GluciQuizz Score	T1D *n* = 66		*p*	T2D *n* = 106		*p*
	≤25 GluciQuizz Score *n* = 35	>25 GluciQuizz Score *n* = 31		≤18 GluciQuizz Score *n* = 55	>18 GluciQuizz Score *n* = 51	
**Total energy intake**, Kcal/d	1556 ± 518	1723 ± 453	0.17	1486 ± 538	1661 ± 434	0.07
Energy from total carbohydrates, %	40 ± 8	37 ± 8	0.11	38 ± 6	38 ± 6	0.87
Energy from simple carbohydrates, %	17 ± 5	16 ± 5	0.68	15 ± 5	15 ± 4	0.79
Energy from protein, %	18 ± 4	18 ± 4	0.53	19 ± 3	19 ± 4	0.38
Average energy from fat, %	42 ± 8	45 ± 7	0.05	43 ± 6	43 ± 5	0.71
Fiber intake, g/d	19.3 ± 7.9	20.3 ± 6.2	0.54	16.9 ± 6.1	19.5 ± 6.6	0.04
**Carbohydrates**						
Average dietary glycemic index	50.8 ± 5.7	49.7 ± 4.8	0.39	51.7 ± 4.1	52.1 ± 4.9	0.71
Dietary glycemic load, g/d	78.6 ± 33.4	81.5 ± 34.2	0.73	72.5 ± 29.2	83.5 ± 28.4	0.05
Carbohydrates from low-glycemic index foods	1 (2.9)	1 (3.2)	>0.9	0 (0.0)	0 (0.0)	-
Carbohydrates from medium-/high-glycemic index foods *	34 (97.1)	30 (96.8)		55 (100.0)	51 (100.0)	
Added sugars, g/d	22.3 ± 20.9	22.2 ± 16.0	>0.9	13.3 ± 12.7	20.0 ± 18.4	0.04
Fruits, g/d	164.6 ± 113.4	175.7 ± 91.7	0.66	191.3 ± 124.9	183.0 ± 103.5	0.71
Sweet/sweetened foods except for fruit, g/d	91.5 ± 67.7	103.0 ± 72.1	0.51	59.8 ± 60.5	88.1 ± 72.3	0.03
100% fruit juice, mL/d	40.7 ± 83.7	24.6 ± 42.2	0.32	29.0 ± 51.2	18.9 ± 49.0	0.30
Sweet/sweetened beverages except for 100% fruit juice, mL/d	24.0 ± 80.2	16.7 ± 68.5	0.69	15.3 ± 45.5	21.1 ± 68.1	0.61

Data are *N* (%) or mean ± standard deviation. * Low glycemic index, 0 to 39; medium glycemic index, 40 to 59; high glycemic index, 60 to 100.

## Data Availability

The raw data supporting the conclusions of this article will be made available by the authors on request, Privacy, ethical.

## Supplementary Materials

The following supporting information can be downloaded at https://www.mdpi.com/article/10.3390/nu18091415/s1, Table S1: Description of participants with basal-bolus treated T1D and T2D.
