# Supplementary material for: Carbohydrate Knowledge in People with Type 1 and Type 2 Diabetes in the NutriNet-Santé Cohort Study
Source: Nutrients. 2026 Apr 29;18(9):1415. doi: 10.3390/nu18091415 (PMC13165393; doi:10.3390/nu18091415)
Supplement: Supplementary file 1 [file nutrients-18-01415-s001.zip › nutrients-4214945-supplementary.pdf]

Table S1. Description of participants with basal-bolus treated T1D and T2D.

General characteristics	Total<br>N= 94 patients	T1D<br>N= 52 patients	T2D<br>N= 42 patients	p	N
**Age,** years	65.4 ± 12.0	62.0 ± 12.7	69.5 ± 9.7	0.002	94
**Sex**, male	38 (40.4%)	15 (28.8%)	23 (54.8%)	0.02	94
**Body mass index,** kg/m^2	27.1 ± 6.8	23.4 ± 5.2	31.6 ± 5.7	<0.001	94
**HbA1c**, %	7.1 ± 0.8	6.9 ± 0.8	7.4 ± 0.8	0.008	84
**GluciQuizz**					
Total 5 domains	21.2 ± 5.3	23.2 ± 5.2	18.6 ± 4.2	<0.001	94
Domain 1	9.9 ± 2.2	10.5 ± 2.3	9.2 ± 1.7	0.002	94
Domain 2	1.8 ± 1.6	2.2 ± 1.7	1.3 ± 1.2	0.004	94
Domain 3	2.8 ± 1.3	3.2 ± 1.1	2.3 ± 1.3	0.001	94
Domain 4	5.3 ± 1.4	5.6 ± 1.1	4.9 ± 1.7	0.01	94
Domain 5	1.3 ± 1.4	1.6 ± 1.5	0.9 ± 1.0	0.008	94

Data are N (%) or mean ± standard deviation
